# Ochratoxin A Degradation and Stress Response Mechanism of *Brevundimonas naejangsanensis* ML17 Determined by Transcriptomic Analysis

**DOI:** 10.3390/foods13233732

**Published:** 2024-11-21

**Authors:** Zitong Zhao, Zehui Niu, Zhihong Liang

**Affiliations:** 1 College of Food Science and Nutritional Engineering, China Agricultural University, Beijing 100083, China; tonyzhao@cau.edu.cn (Z.Z.); hyeu2023@outlook.com (Z.N.); 2 The Supervision, Inspection and Testing Center of Genetically Modified Organisms, Ministry of Agriculture, Beijing 100083, China; 3 Beijing Laboratory for Food Quality and Safety, College of Food Science and Nutritional Engineering, China Agricultural University, Beijing 100083, China

**Keywords:** *Brevundimonas naejangsanensis* ML17, ochratoxin A, transcriptomic analysis, stress response, anti-oxidative stress, detoxifying enzyme, degradation mechanism

## Abstract

Ochratoxin A (OTA) is a naturally occurring mycotoxin mainly produced by certain species of *Aspergillus* and *Penicillium* and is a serious threat to human health and food safety. Previous studies showed that *Brevundimonas naejangsanensis* ML17 can completely degrade 1 μg/mL of OTA. The aim of this study was to investigate the degradation effect of ML17 at different concentrations of OTA, and specifically, to investigate the mechanism of OTA degradation by ML17. The growth of ML17 was not affected by exposure to 6 μg/mL OTA within 24 h. ML17 could almost completely degrade 12 μg/mL of OTA within 36 h, converting it into the non-toxic OTα and L-phenylalanine. Transcriptomic analysis showed that 275 genes were upregulated, whereas three genes were downregulated in ML17 under the stress of 1 μg/mL OTA. Functional enrichment analysis showed that exposure to OTA enhanced translation, amide and peptide biosynthesis and metabolism, promoted oxidative phosphorylation, and increased ATP production. Further analysis revealed that, when exposed to OTA, ML17 exerted a stress-protective effect by synthesizing large amounts of heat shock proteins, which contributed to the correct folding of proteins. Notably, genes related to antioxidant activity, such as peroxiredoxin, superoxide dismutase, and glutaredoxin 3, were significantly upregulated, indicating that ML17 can resist the toxic effects of OTA through adjusting its metabolic processes, and the enzyme-coding *gene0095*, having OTA degradation activity, was found to be upregulated. This suggests that ML17 can achieve OTA degradation by regulating its metabolism, upregulating its antioxidant system, and upregulating enzyme-encoding genes with OTA degradation activity. Our work provides a theoretical reference for clarifying the mechanism of OTA degradation by ML17.

## 1. Introduction

Mycotoxins are a group of secondary metabolites produced by mycotoxigenic fungi, which are prevalent in many food products and nuts [1]. Ochratoxins (OTs) are naturally occurring mycotoxins produced by certain *Aspergillus* spp. and *Penicillium* and mainly include ochratoxin A (OTA), ochratoxin B, ochratoxin C, and other derivatives [2]. Ochratoxin A, a highly toxic substance discovered in 1969, is widely distributed, and the complete control of mycotoxins and mycotoxigenic fungi cannot be achieved throughout the entire long chain of farm to fork. According to the European Union Rapid Alert System (RASFF) for Food and Feed notification, a high detection rate of OTA in grapes, coffee, and crops has a serious negative impact on food safety. Therefore, OTA-contaminated foods and food products cannot be processed and consumed normally, resulting in huge economic losses [3,4]. Currently, detoxification of contaminated food and food ingredients is extremely important to ensure food safety and reduce economic losses caused by mycotoxin contamination [5].

Several physical and chemical methods have been explored to reduce mycotoxins in food and food ingredients [6]. These traditional methods may have the disadvantages of low detoxification efficiency, affecting the flavor and quality of food and introducing new harmful substances, which is not ideal for eliminating mycotoxin contamination [7,8]. Biodegradation methods utilize adsorption by the microorganism’s cell wall and the degradation activity of intracellular enzymes to achieve the efficient removal of mycotoxins [9,10]. Benefitting from greener, safer, and more efficient characteristics than traditional methods, this method has been recognized as most promising for solving mycotoxin contamination and has become a popular topic in current research.

The degradation of OTA by microorganisms involves a series of complex processes involving a variety of degradation mechanisms such as cell wall adsorption, metabolic digestion, and degradation by secreted enzymes, intracellular enzymes, and peptides [5,11]. However, the microbial detoxification function is unstable, and after multiple generations of culture, the degradation activity may be reduced or eliminated, hindering the development and application of microbial agents for OTA degradation [12]. OTA-degrading enzymes mined from functional strains can be prepared in large quantities using chassis biology and cell factories, and their mycotoxin-degrading activity is highly efficient and stable [13,14]. Therefore, quickly identifying functional factors, including enzymes and peptides, capable of efficiently degrading OTA from functional microorganisms is key to breaking the barriers to mining mycotoxin-degrading functional factors. Transcriptomic analysis can be used to compare differences in gene expression of microorganisms in the presence of mycotoxin stress, which could assist in the mining of mycotoxin-degrading functional factors, especially degradative enzymes, by analyzing the microbial response to toxin stress and the expression of related detoxification genes [15,16,17].

Bioenzymes have been isolated from various bacteria, fungi, and yeasts identified with OTA degradation activity [9,18]. Our previous research isolated a bacterial strain from soil that was identified by whole genome sequencing as *Brevundimonas naejangsanensis* ML17, and it could degrade OTA into OTα by a degradation rate of 100% at 37 °C within 24 h [19]. Meanwhile, the OTA removal of its inactivated and uninactivated strains was investigated, and the results showed that the OTA removal efficiency of the inactivated strains was much lower than that of the uninactivated strains, suggesting that the OTA removal activity of this strain mainly originated from its degrading effect [19]. Subsequently, four enzymes with OTA degradation activity, *gene0095*, *gene1826*, *gene2253*, and *gene0484*, were isolated and purified from the ML17 strain. However, we found that the degradation activity of viable bacterial cells and their intracellular components on OTA was consistently stronger than that of the identified enzymes, suggesting that other enzymes or peptides may be involved in the degradation of OTA by *B. naejangsanensis* ML17 or a synergistic effect of multiple components [2]. Therefore, the intrinsic mechanism of OTA degradation by *B. naejangsanensis* ML17 requires further investigation. Messenger RNA (mRNA) transcribed from DNA regulates cellular activity by controlling protein biosynthesis. Transcriptome analysis has been shown to be effective for RNA sequencing, valuable for discovering functional enzymes, and is currently being used to study changes in gene expression and detoxification mechanisms in the basidiomycetous yeast *Apiotrichum mycotoxinivorans* exposed to OTA [20]. Hence, the purpose of our study was to determine whether treatment with OTA altered gene expression in *B. naejangsanensis* ML17 and thereby reveal its detoxification mechanism.

Based on previous studies [2,19], the present study mainly investigated the growth and OTA degradation ability of *B. naejangsanensis* ML17 under different levels of OTA stress and then analyzed the mechanism of synergistic bacterial–enzymatic OTA degradation through comparative transcriptomic analysis under OTA stress. Our results provide a basis for the application of mycotoxin-degrading bacteria and the prediction or validation of mycotoxin detoxification enzymes.

## 2. Materials and Methods

### 2.1. Chemicals and Regents

Ochratoxin A (purity ≥ 99%) was purchased from Pribolab Pte., Ltd. (Qingdao, China). Yeast extract and tryptone was supplied by Oxoid (London, UK). Silica gel plates for thin-layer chromatography (G type) were provided by Dingkang Silica Co., Ltd. (Qingdao, China). Ethanol, ethylacetate, formic acid, and acetonitrile were purchased from Macklin Inc. (Shanghai, China). Toluene, trichloromethane, and hydrochloric acid were supplied by Sinopharm Chemical Reagent Co., Ltd. (Shanghai, China).

### 2.2. Strains and Culture Conditions

*B. naejangsanensis* ML17 was isolated by our laboratory and preserved at the China General Microbiological Culture Collection Center (CGMCC No. 22280). The method for the preparation of Luria-Bertani (LB) medium was to first mix 1 g of tryptone, 0.5 g of extract of yeast and 1 g of NaCl, adjust the pH to 7.0 and then dilute with distilled water to a final volume of 100 mL. Finally, LB medium was sterilized under high pressure (121 °C, 15 min). The solid medium was supplemented with agar (1.5 g/100 mL). *B. naejangsanensis* ML17 strain was inoculated into LB liquid medium and incubated at 37 °C for 24 h and then spread onto a plate and cultured at 37 °C for 24 h. A single colony inoculated into LB liquid medium for 3–4 generations of growth.

### 2.3. Growth Curve Measurements of B. naejangsanensis ML17 Under OTA Stress

In order to investigate the growth of *B. naejangsanensis* ML17 under different levels of OTA stress, the strain was inoculated into 100 mL of LB medium (1% inoculum), incubated at 37 °C and 200 rpm. Our previous study showed that the ML17 strain could grow rapidly at 37 °C, so the subsequent culture temperature of the strain and the determination of OTA degradation activity were also measured at this temperature [19]. Simultaneously, OTA was added at final concentrations of 1, 2, 6, and 12 μg/mL, respectively, and the sample without OTA was used as the control group. The OD_600_ values of the samples were determined every 2 h by UV-visible spectrophotometer (UV-2102PC, Unico Instrument, Shanghai, China) and the growth curves of the strains were plotted according to the measured values. Meanwhile, the samples with corresponding time were retained for the subsequent determination of the OTA degradation rate.

### 2.4. Morphological Determination of B. naejangsanensis ML17

*B. naejangsanensis* ML17 was coated on LB plates and incubated upside down at 37 °C for 2 d. Colony morphology and color were observed and photographed. Gram staining was performed by adding 10 μL bacterial solution to a glass slide and uniformly coating, fixing with an alcohol lamp flame, staining with crystal violet and iodine solution for 1 min each, decolorizing with 95% ethanol for 30 s, and re-staining with safranine for 2 min. In addition, *B. naejangsanensis* ML17 cultured to logarithmic growth stage was fixed with 2.5% glutaraldehyde to prepare samples for scanning electron microscopy. Finally, the cell morphology of *B. naejangsanensis* ML17 was examined using a scanning electron microscope (Zhongke Baijie Company, Beijing, China).

### 2.5. Detection of OTA-Degrading Activity of B. naejangsanensis ML17

#### 2.5.1. Detection OTA-Degrading Activity of *B. naejangsanensis* ML17 Using Thin-Layer Chromatography (TLC)

The detection method was developed by Peng et al. [2]. *B. naejangsanensis* ML17 (300 μL) was reacted with different concentrations of OTA (0, 1, 2, 6, and 12 μg/mL) at 37 °C for different times (6, 12, 18, 24, 36, and 60 h). On completion of the reaction, 10 μL of 6 mol/L HCl solution was added to make the pH 2–3. Then, 100 μL of CHCl_3_ was added, vortexed, mixed, and centrifuged (12,000× *g*, 2 min). In total, 10 μL of the bottom layer was placed on a high-performance G-type silica gel plate for thin-layer chromatography. The sampled silica gel plate was placed on a chromatography cylinder for chromatographic analysis. Toluene, ethyl acetate and formic acid were mixed in a volume ratio of 6:3:1 and used as solvents. The plates were developed until the solvent reached 2/3 of the silicone plate from the point of application along the front line, and the developer was recovered. After drying the silicone plate using a hair dryer, it was observed and photographed under UV light (365 nm). OTA emits blue-green fluorescence and the retention factor value (Rf) is approximately 0.65.

#### 2.5.2. Detection OTA-Degrading Activity of *B. naejangsanensis* ML17 Using High-Performance Liquid Chromatography (HPLC)

The amount of OTA was detected by using the HPLC system (SPD-20A; Shimadzu, Kyoto, Japan) and in accordance with the previously published article [2]. After the reaction with OTA, 300 μL of the components was extracted with CHCl_3_ to extract the OTA and its degradation products in the reaction system. CHCl_3_ was extracted twice, then the CHCl_3_ extract was combined, blown dry, dissolved in 300 μL of methanol, and filtered through a 0.22 μm organic microporous filter membrane. The parameters of HPLC were as follows: Spherisorb S5 ODS2 C18 column (Waters Corporation, Milford, CT, USA), 150 × 4.6 nm, i.d. 3.5 mm. Mobile phase A was 0.1% volume of formic acid, and mobile phase B was acetonitrile containing 0.1% volume of formic acid. The elution mode was an isocratic gradient, the total flow rate was 1 mL/min (0.3 mL/min for phase A, 0.7 mL/min for phase B), and the injection volume was 10 μL. The fluorescence detector was operated at an excitation wavelength of 333 nm and an emission wavelength of 460 nm, and the results were analyzed by Shimadzu LabSolutions Ver. 5 software. The degradation rate was calculated as OTA degradation rate = (1 − OTA peak area in treatment/peak area in control) × 100%.

### 2.6. Transcriptomic Analysis

Transcriptome analysis was reference to Yang et al. [15] and entrusted to Novogene Co., Ltd. (Beijing, China) for library construction. The *B. naejangsanensis* ML17 was inoculated into 100 mL of LB medium (1% inoculum), and the final concentration of 1 μg/mL OTA was added and incubated at 37 °C and 200 rpm for 24 h. The cultures with equal amounts of methanol were used as the control group under the same conditions, and three parallel groups were formed in each group. After incubation, the bacterial solution was centrifuged at 4 °C and 8000× *g* for 10 min, the supernatant was discarded, and the bacterial precipitate was transferred to a 1.5 mL sterile freezing tube and stored in a −80 °C refrigerator. The total RNA in *B. naejangsanensis* ML17 was extracted by Invrogen TRIzol, and the concentration and integrity of RNA were accurately detected by Agilent 2100 bioanalyzer (Agilent Technologies, Santa Clara, CA, USA). cDNA library construction and mRNA sequencing were carried out by Novogene Co., Ltd. (Beijing, China). Analytical tools used were RSEM (http://deweylab.github.io/RSEM/, accessed on 2 June 2024), Kallisto (https://pachterlab.github.io/kallisto/, accessed on 5 June 2024), and Salmon (https://combine-lab.github.io/salmon/, accessed on 5 June 2024).

### 2.7. Functional Annotation and Enrichment Analysis

In order to investigate the differential expression gene (DEG) in *B. naejangsanensis* ML17 under OTA stress, gene ontology (GO) annotate, and Kyoto Encyclopedia of Genes and Genomes (KEGG) for gene function definition were used to analyze the DEGs according to previous references [15]. GOatools (https://github.com/tanghaibao/GOatools, accessed on 25 June 2024) was used to identify significantly enriched GO terms using Fisher’s exact test. GO terms with adjusted *p* < 0.05 were considered significantly enriched DEGs.

### 2.8. Statistics

All data plots in this paper were generated using Prism 9 (GraphPad Software, Inc. San Diego, CA, USA). All data were subjected to Duncan’s multiple comparison test and differences were considered significant at *p* < 0.05. One-way analysis of variance (ANOVA) was performed on the data using SPSS (version 20.0, Chicago, IL, USA).

## 3. Results

### 3.1. Growth of B. naejangsanensis ML17 under Different Levels of OTA Stress

**Figure 1:**
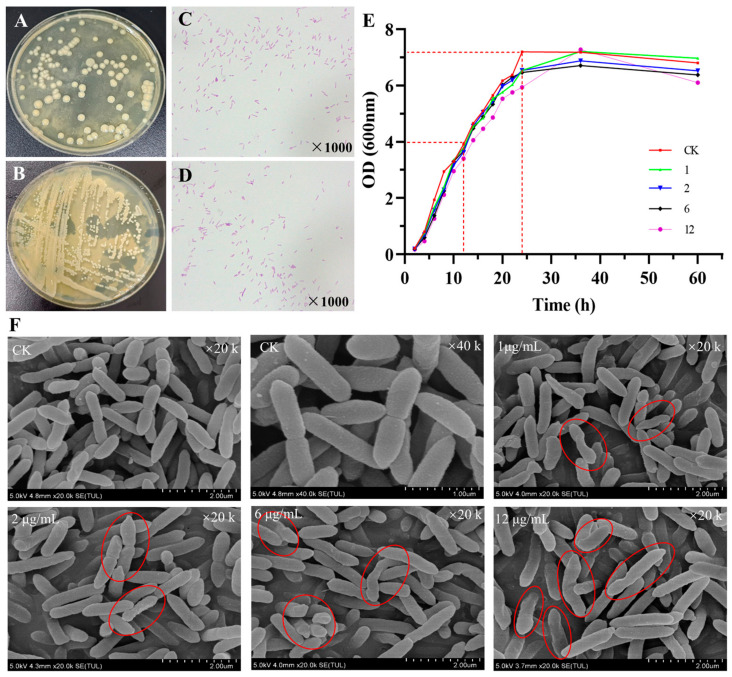
Morphology ((**A**,**B**) colony; (**C**,**D**) optical microscope) and growth curves of *B. naejangsanensis* ML17 under different OTA concentrations (**E**); (**F**) Scanning electron microscopy image of *B. naejangsanensis* ML17 morphology at different OTA concentrations. CK, 1, 2, 6, and 12 represent the growth curve of *B. naejangsanensis* ML17 when exposed to 0, 1, 2, 6, and 12 μg/mL OTA, respectively. Red marker shows cells with abnormal and distorted morphology.

The colony morphology of *B. naejangsanensis* ML17 after activation culture on agar plates is shown in Figure 1A,B; *B. naejangsanensis* ML17 colonies are shiny with smooth and clean edges. Optical microscope observations after Gram staining showed Gram-negative bacteria in the form of rods (Figure 1C,D). Scanning electron microscopy showed asymmetric cell division and the size of the bacterium was about 0.3 × (1–2) μm. These traits are consistent with the description of this bacterium in Bergey’s Manual of Systematic Microbiology [21]. The growth curve of *B. naejangsanensis* ML17 in the presence of OTA (1, 2, 6, and 12 μg/mL) is shown in Figure 1E. The results show that within 24 h, OTA concentrations below 6 μg/mL did not affect the growth of ML17. However, an inhibitory effect on *B. naejangsanensis* ML17 growth was observed after 24 h, with the final density of ML17 decreasing with increasing OTA concentration. At 12 μg/mL of OTA, *B. naejangsanensis* ML17 growth was inhibited from the beginning of cultivation.

The morphology of *B. naejangsanensis* ML17 at different OTA concentrations (1, 2, 6, and 12 μg/mL) is shown in Figure 1F. Compared to the control group (CK), which was not exposed to OTA, the number of bacterial morphological distortions gradually increased with increasing OTA concentration, indicating that exposure to OTA causes abnormal morphological distortions in some bacterial cells. However, owing to its ability to degrade OTA, the growth of this strain was not completely inhibited, even in the presence of high concentrations of OTA. Specific intracellular pathways may have been triggered to counteract the effects of environmental toxins under OTA stress or specific degrading enzymes produced to degrade toxins in the environment, thereby improving the stress resistance of the strain and adapting to survive in high OTA environments. Therefore, analyzing the transcription process under OTA stress, studying the specific processes activated in response to OTA stress, and exploring the possible OTA-degrading enzymes involved are necessary.

### 3.2. Degradation of OTA by B. naejangsanensis ML17 Under OTA Stress

**Figure 2:**
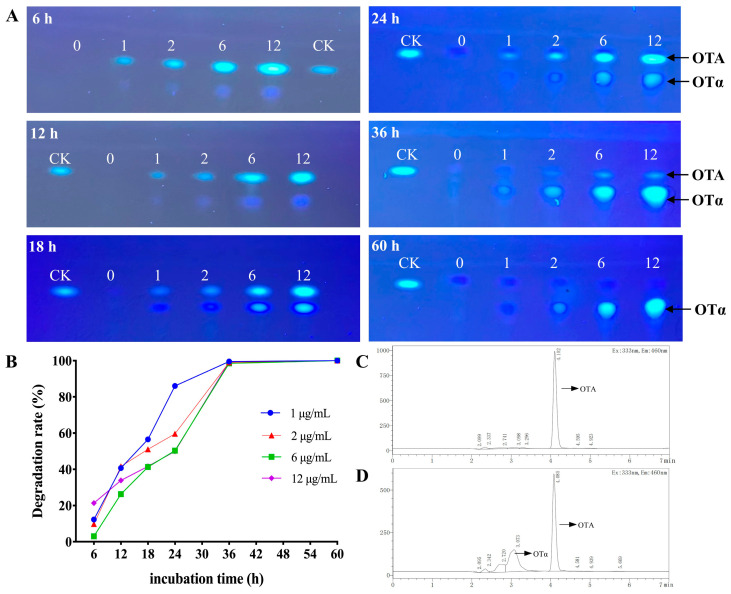
(**A**) Degradation of OTA by *B. naejangsanensis* ML17 at different OTA concentrations detected by thin-layer chromatography (TLC); (**B**) OTA degradation rate detected by HPLC; (**C**) HPLC profile of OTA; (**D**) HPLC profile showing the OTA-degrading activity of ML17. 0, 1, 2, 6, and 12 represent *B. naejangsanensis* ML17 treated with 0, 1, 2, 6, and 12 μg/mL OTA, respectively; CK indicates only OTA.

The degradation of OTA by *B. naejangsanensis* ML17 under different levels (0, 1, 2, 6, and 12 μg/mL) of OTA stress and different cultivation times (6, 12, 18, 24, 36, and 60 h) is shown in Figure 2. These results indicate that *B. naejangsanensis* ML17 can degrade OTA at different concentrations. After 24 h co-culturing of *B. naejangsanensis* ML17 with 6 and 12 μg/mL OTA, approximately 50% of OTA was degraded which was detected by HPLC; co-culturing of *B. naejangsanensis* ML17 with 1, 2, 6, and 12 μg/mL OTA for 36–60 h, resulted in complete degradation of OTA (Figure 2B). Results of HPLC detection of OTA and confirmation of its degradation product OTα are shown in Figure 2C,D, respectively. The HPLC results demonstrated that *B. naejangsanensis* ML17 can efficiently degrade the amide bonds of OTA and convert OTA into OTα and L-phenylalanine.

### 3.3. Transcriptome Features Under OTA Exposure

**Figure 3:**
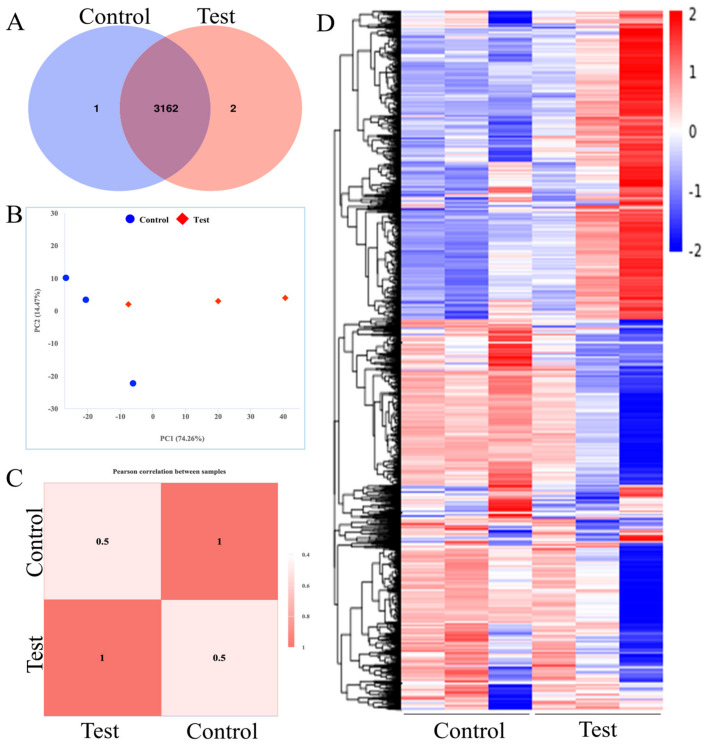
Gene expression in *B. naejangsanensis* ML17. (**A**) Venn diagram of gene expression changes between control and test. (**B**) Control and test group gene expression PCA analysis. (**C**) Gene expression in control and test groups by Pearson correlation analysis. (**D**) Cluster heatmap analysis of gene expression levels between the control and test groups. Control, without OTA; Test, under 1 μg/mL OTA stress.

We performed transcriptome sequencing after culturing *B. naejangsanensis* ML17 for 24 h with 0 μg/mL and 1 μg/mL OTA (control and test group, respectively). The results showed that the control and test groups expressed 3163 and 3164 genes, respectively, of which 3162 were co-expressed (Table S1). The number of genes expressed between the two groups did not differ, indicating that OTA had no effect on the type or number of genes expressed in *B. naejangsanensis* ML17 (Figure 3A). PCA cluster analysis of gene expression in the control and test groups revealed significant differences in clustering (Figure 3B) with a Pearson correlation coefficient of 0.5 (Figure 3C), indicating that exposure to 1 μg/mL OTA significantly altered the expression levels of genes in *B. naejangsanensis* ML17. Next, we performed a cluster heat map analysis of the 3162 genes expressed in the control and test groups (Figure 3D). We found that exposure to 1 μg/mL OTA has a substantial effect on the expression level of genes in *B. naejangsanensis* ML17. Exposure to OTA markedly upregulated or downregulated the expression of certain genes in *B. naejangsanensis* ML17.

**Figure 4:**
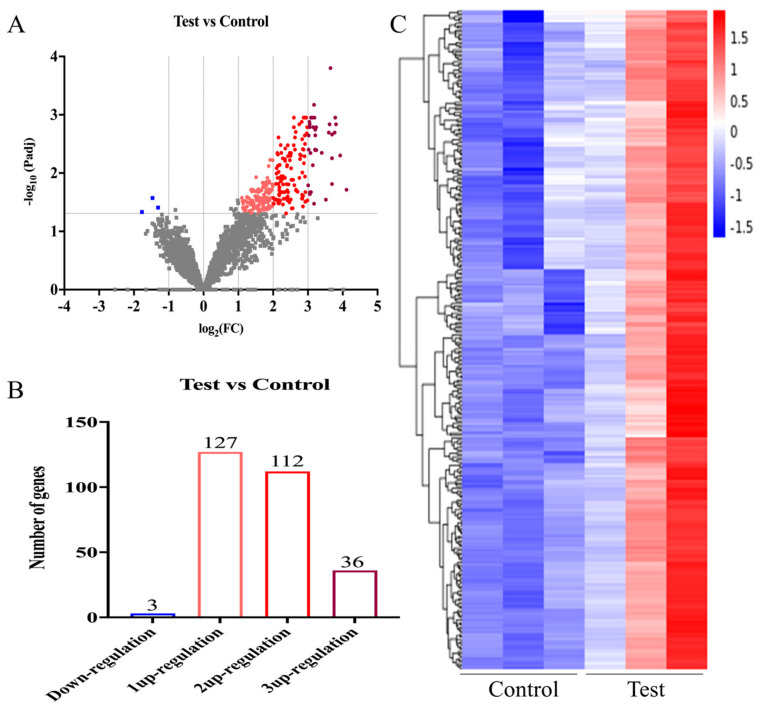
Effect of OTA exposure on gene expression in *B. naejangsanensis* ML17. (**A**) Volcano plot analysis of differentially expressed genes between the control and test groups; Gray: no significant difference in gene expression; blue: significant decrease in gene expression, rose: 3up-regulation in gene expression; red: 2up-regulation in gene expression; pink: 1up-regulation in gene expression. (**B**) Number of differentially expressed genes between the control and test groups. (**C**) Cluster heatmap analysis of differentially expressed genes between the control and test groups. Control, without OTA; Test: under 1 μg/mL OTA stress.

The differentially expressed genes between the control and test groups are shown in Figure 4. Under exposure to 1 μg/mL OTA, the expression levels of 2886 genes in *B. naejangsanensis* ML17 did not change significantly; however, a significant effect was observed on the expression levels of 278 genes (Table S2). Of these, three genes were significantly downregulated. In contrast, 275 genes were significantly upregulated (Figure 4A) (*p* < 0.05). Among the 275 significantly upregulated genes, the expression levels of 36 genes were 3-fold those of the control group, and that of 112 genes was 2-fold those of the control group (Figure 4B) (*p* < 0.05). The results of the study showed that 1 μg/mL OTA significantly increased the level of gene expression (*p* < 0.05), although it had no effect on the number and type of *B. naejangsanensis* ML17 genes expressed.

### 3.4. Functional Enrichment Analysis of Differentially Expressed Genes

**Figure 5:**
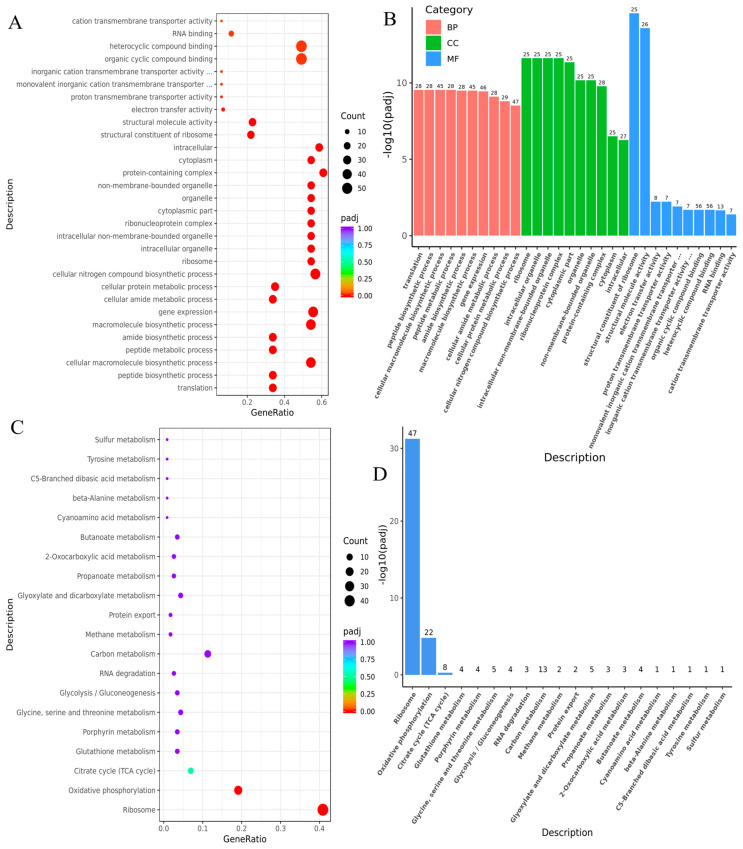
Functional analysis of differentially expressed genes in *B. naejangsanensis* ML17 between the control and test groups. (**A**) Gene ontology (GO) enrichment dot bubble plot of differentially expressed genes. (**B**) GO enrichment terms of differentially expressed genes. (**C**) Kyoto Encyclopedia of Genes and Genomes (KEGG) enrichment dot bubble plot of differentially expressed genes. (**D**) KEGG enrichment terms of differentially expressed genes.

To investigate the involvement of differentially expressed genes in the biological processes of *B. naejangsanensis* ML17, we performed GO (Figure 5A,B) and KEGG (Figure 5C,D) functional enrichment analyses of the 275 genes whose expression levels were significantly increased under OTA stress. We found that the differentially expressed genes upregulated by OTA were markedly accumulated in 55 GO terms, containing 23 biological processes, 13 cellular components, and 19 molecular functions (Table S3). The 30 most significantly enriched GO terms included 10 biological processes, 10 cellular components, and 10 molecular functions (Figure 5A,B). Exposure to OTA promoted translation (GO: 0006412) and gene expression (GO: 0010467) in *B. naejangsanensis* ML17, consistent with the observation that more genes were upregulated than downregulated upon exposure to OTA. This suggests that under OTA stress, *B. naejangsanensis* ML17 improves its translation efficiency to synthesize more proteins. In addition, the enrichment of processes such as peptide biosynthesis (GO:0043043), peptide metabolism (GO:0006518), cellular protein metabolism (GO:0044267), organonitrogen compound biosynthesis (GO:1901566), and protein metabolism (GO:0019538) (Table S3) indicated that under OTA stress, *B. naejangsanensis* ML17 promoted protein and peptide synthesis and metabolism.

Furthermore, GO terms related to the oxidative phosphorylation process included oxidation reduction (GO: 0055114) and electron transfer activity (GO: 0009055). Electron transfer activity is related to all the molecular entities that serve as electron donors and acceptors in the electron transport chain. Most ATP produced during the aerobic catabolism of glucose is obtained from this electron transfer chain process [22], suggesting an increase in ATP production. Consistent with the GO enrichment results, the differentially expressed genes upregulated upon exposure to OTA were significantly enriched in two KEGG pathways: ribosome (*bne03010*) and oxidative phosphorylation (*bne00190*) (Figure 5C,D). We conclude that OTA exposure promoted translation in *B. naejangsanensis* ML17, increased aerobic respiration and ATP production, and promoted the biosynthesis and metabolism of peptides, amides, proteins, biomolecules, and organic nitrogen compounds.

### 3.5. Stress Response of B. naejangsanensis ML17 to OTA

Heat shock proteins (HSPs), a family of chaperone proteins, are a class of proteins produced by cells under stress conditions. By helping to correctly fold newly synthesized proteins or refold stress-damaged proteins, HSPs help proteins form the correct three-dimensional structure and correct any inappropriate hydrophobic structures. GroES is an ATP-dependent chaperone protein that prevents protein aggregation and promotes protein solubility, making it a typical component for soluble protein folding. In addition, GroEL promotes the folding and assembly of membrane proteins. The protease ClpP consists of ClpP protease subunits and Clp ATP-binding subunits (ClpA/ClpX). ClpP plays an important role in maintaining normal metabolism and adapting to various stress responses in the body by using the energy generated by ATP to act as a proteolytic enzyme that degrades damaged proteins. ClpB is a member of the ClpP family of proteases that can break down protein aggregates and repair denatured proteins synergistically with other companion proteins. As shown in Table 1, our study found that post OTA exposure, *B. naejangsanensis* ML17 increased the gene expression sites of HSPs, including IbpA and DnaK, the alpha crystallin family, GroEL, GroES, ClpB, ClpP, ClpA, and ClpX, stress-induced and universal stress proteins, and the GlsB/YeaQ/YmgE family stress response membrane proteins. Our results suggest that OTA exposure leads to protein misfolding, resulting in intracellular stress responses. *B. naejangsanensis* ML17 synthesizes many HSPs and stress-induced proteins to guide and repair the correct folding of proteins, break down protein aggregates, and resist the stress response caused by OTA.

### 3.6. Effect of OTA on Transport Proteins in B. naejangsanensis ML17

The TonB system in bacteria is crucial for the uptake of essential nutrients from the environment. The TonB system consists of the inner membrane-anchored ExbB-ExbD and the peripheral protein TonB (TonB-ExbB-ExbD, Ton complex) that provide energy to TonB-dependent outer membrane receptors (TBDTs), enabling them to specifically transport relatively large compounds and nutrients. In addition, the TBDT and Ton complexes are essential for the uptake of lignin-derived aromatic compounds by the outer membrane [23]. As shown in Table 2, our research indicated that under OTA stress, the expression of seven TonB system gene loci on the outer membrane of *B. naejangsanensis* ML17 increased, specifically those transporting and promoting the absorption of iron carrier compounds, vitamin B12, and transition metal elements such as copper, zinc, and nickel. Iron ions are essential nutrients for almost all organisms, including pathogenic bacteria. Iron ions form the catalytic centers of important biological enzymes such as oxidoreductases, which promote various life activities including electron transfer, antioxidant reactions, and nucleic acid synthesis. Vitamin B12 is an important micronutrient for bacteria and is involved in the synthesis of various biological enzymes. Increased gene expression of periplasmic copper chaperone A also indicates increased intracellular copper transport activity. In addition, under OTA stress, the expression of porin family proteins increased in *B. naejangsanensis* ML17. Compared with ion channels, porins have low selectivity and are responsible for the non-specific passive transport of nutrients. Our study also found increased gene expression of the putative sulfate export family of transporter proteins, which promote sulfate transport and efflux from cells.

Mechanosensitive ion channels are membrane-embedded polymeric proteins that mediate the transmembrane ion transport. In the kidney, OTA uptake by cells depends on organic anion transporter proteins [24,25]. Research shows that OTA is cleared from cells through the transport of human breast cancer resistance protein and multidrug resistance protein 2, reducing the toxicity of OTA [26]. Low-dose OTA leads to increased expression of organic anion transport proteins in rat kidneys [27]. Our present study suggests that, under OTA stress, the expression of mechanosensitive ion channels in the outer membrane of *B. naejangsanensis* ML17 increases, which may be involved in the transport of OTA by cells. Our research indicates that under OTA stress, *B. naejangsanensis* ML17 enhances nutrient uptake by increasing the expression of membrane transport proteins such as the TonB system, porin family of proteins, sodium solute symporter family, mechanosensitive ion channel, vitamin B12 and iron complex, and polar copper chaperone A.

### 3.7. Effect of OTA on Antioxidant Levels in B. naejangsanensis ML17

In addition to protein damage, OTA causes lipid peroxidation, increases reactive oxygen species (ROS) and malondialdehyde levels, and consumes endogenous antioxidants, leading to oxidative stress [28]. Reactive oxygen species (ROS) can be catalyzed into H_2_O_2_ by superoxide dismutase (SOD). Our study found that *B. naejangsanensis* ML17 significantly upregulated two SOD gene loci (Table 3), one of which belongs to the Fe-Mn SOD family. Depending on the catalyst used, SOD has been classified into the Cu-Zn SOD, Fe-Mn SOD, and rare Ni SOD families [29]. *B. naejangsanensis* ML17 inhibits oxidative stress caused by OTA by upregulating the expression of Fe-Mn SOD, which protects cells from damage. O^2−^ is converted to H_2_O_2_ by SOD. However, in the presence of Fe^2+^, H_2_O_2_ can be converted to the very reactive hydroxyl radical (^•^OH), which can attack various biomolecules. Glutathione peroxidase (GSH-Px) and peroxidase convert H_2_O_2_ to H_2_O and O_2_.

Our study found that after OTA exposure, one glutathione (GSH)-Px gene locus, one glutathione S-transferase gene locus, one hydroxyacylglutathione hydrolase gene locus, and two GSH gene loci (GSH3 and monothiol GSH) were significantly upregulated in *B. naejangsanensis* ML17 (Table 3). Glutathione is a major antioxidant that helps to scavenge ROS and preserve the redox balance. It acts as an electron donor to catalyze the reduction of H_2_O_2_ by GSH-Px. In addition, GSH is also involved in cellular detoxification. Glutathione detoxifies organisms through glutathione S-transferase, which catalyzes the reaction of GSH with compounds containing thiols, double bonds, halogens, and other functional groups, thereby promoting its excretion. Multidrug-resistance-related proteins that have been functionally characterized to date have the property of being ATP-dependent efflux pumps that are used to transport GSH S conjugates and can also excrete conjugates with glucuronides or sulfates from cells. Hydroxyl glutathione hydrolase is also a reactive biological detoxification enzyme. Ochratoxin A is biotransformed into an active intermediate, which reacts covalently with GSH to form a conjugate. Ochratoxin A and GSH conjugates (OT-GSH) have been detected in animal and human tissues, blood, and urine [30,31].

Peroxidases are a rich family of thiol-dependent peroxidase enzymes that decompose H_2_O_2_ and have a central function in maintaining cellular responses to alterations in redox homeostasis. The present study found that two peroxidase gene loci were significantly upregulated in *B. naejangsanensis* ML17 (Table 3). Peroxiredoxin Q/BCP and peroxiredoxin (alkyl hydrogen peroxide reductase subunit C) belong to the alkyl hydrogen peroxide reductase family and possess antioxidant activities. Previous studies have shown that peroxidases can degrade OTA. Nora et al. [32] reported that commercial peroxidase and peroxidase extracted from rice had OTA degradation rates of 41% and 59%, respectively. Horseradish peroxidase has also been reported to degrade OTA with an OTA degradation rate of 27% in the presence of a H_2_O_2_ reaction system [33].

Our research suggests that, in response to OTA-induced oxidative stress, *B. naejangsanensis* ML17 scavenges intracellular ROS by secreting SOD, GSH-Px, and peroxiredoxin. Intracellular detoxification processes may also be enhanced by secreting GSH and glutathione S-transferase, thereby repairing cellular damage.

### 3.8. Effect of OTA on the Levels of Active Enzymes in B. naejangsanensis ML17

The enzymes that have been reported to degrade OTA include carboxypeptidases, amidases, and proteases. The degradation mechanism of carboxypeptidases is to hydrolyze the lactam bond of the OTA molecule to produce OTα and phenylalanine. We first analyzed the changes in the expression of four hydrolytic enzymes, *gene0095*, *gene1826*, *gene2253*, and *gene0484*, secreted by *B. naejangsanensis* ML17 when exposed to OTA. A previous study has reported that these four hydrolases isolated from the extracellular fluid of *B. naejangsanensis* ML17 can degrade OTA to produce OTα and phenylalanine [2]. As shown in Table 4, only the expression level of *gene0095* was significantly increased two-fold under OTA stress, and the expression levels of *gene1826*, *gene2253*, and *gene0484* were not significantly different from those in the control group (*p* < 0.05). In addition, we found four other enzymes significantly expressed by *B. naejangsanensis* ML17 under OTA exposure that may have OTA degradation activity, namely *gene1837* (leucinamide peptidase), *gene1744* (peptidase), *gene1792* (serine hydrolase), and *gene1036* (the serine endopeptidase/trypsin-like peptidase family).

## 4. Discussion

OTA is a class IIB carcinogen with teratogenic, carcinogenic, and mutagenic effects, which have serious impacts on food safety [34]. The mechanism of OTA toxicity may involve the generation of ROS, which causes structural damage and functional disorders in the mitochondria, leading to the upregulation or downregulation of a series of physiological and biochemical mediators. Although the biotransformation mechanism of OTA-induced ROS generation is still not well understood, ROS is generally accepted as playing an important role in OTA toxicity.

In this study, we observed that *B. naejangsanensis* ML17 was extremely tolerant to OTA and degraded it efficiently; 12 μg/mL of OTA was completely degraded within 36 h. Consistent with the results of a previous study [19], our present study demonstrated that *B. naejangsanensis* ML17 can efficiently degrade the amide bonds of OTA and convert OTA into OTα and L-phenylalanine. Ochratoxin α is much less toxic than OTA and can be considered almost non-toxic. From the viewpoint of degradation products, *B. naejangsanensis* ML17 degrades OTA to OTα, and this is currently recognized as the best degradation method to solve the problem of OTA pollution. High-performance liquid chromatography analysis of the degradation products showed that *B. naejangsanensis* ML17 could degrade OTA (RT = 4.10) to OTα (RT = 3.07); thus, we concluded that *B. naejangsanensis* ML17 may express one or more enzymes with OTA degrading activity.

However, the detoxification mechanism remains unknown, thus restricting its wider application. Ochratoxin A exposure affects various bacteria differently and their detoxification efficiencies, which rely on their specific detoxification mechanisms, differ significantly [35]. Two pathways are hypothesized for the biodegradation of OTA. The first pathway involves irreversible hydrolysis of the amide bond of the OTA and the second pathway involves reversible hydrolysis of the lactone ring [36]. Previous studies by our team have shown that *B. naejangsanensis* ML17 secretes four carboxypeptidases with OTA degradation activity, *gene0095*, *gene1826*, *gene2253*, and *gene0484*, into the extracellular compartment. However, we found that the intracellular fraction and viable bacteria consistently showed stronger OTA degradation activity than the identified enzymes, suggesting that other components may be important in OTA degradation by *B. naejangsanensis* ML17 or a synergistic effect of multiple components [2].

Therefore, we utilized RNA-Seq to investigate *B. naejangsanensis* ML17’s response to OTA and further elucidate its detoxification mechanism. Comparative transcript analysis revealed that 275 genes were upregulated and three genes were downregulated in *B. naejangsanensis* ML17 in response to stimulation with 1 μg/mL OTA. Functional classification and enrichment analyses revealed that OTA treatment increased gene expression, translation, amide and peptide biosynthesis, peptide and protein metabolism, and oxidative phosphorylation, leading to increased protein synthesis and catabolism. Further analyses showed that when exposed to OTA, *B. naejangsanensis* ML17 exerted a stress-protective effect by synthesizing large amounts of HSPs, which contributed to the correct folding of the proteins. In addition, the oxidation-reduction (GO:0055114) and electron transfer activity (GO:00009055) pathways associated with the oxidative phosphorylation ATP-producing process were also significantly upregulated, suggesting that ATP production was increased and aerobic respiration was enhanced, which in turn promoted the translation level of *B. naejangsanensis* ML17.

An important reason for the cytotoxicity of OTA is that it induces excessive production of ROS in cells [37,38]. Reactive oxygen species as signaling molecules can induce the production of various antioxidants and protective enzymes, which is consistent with the results of this study [39]. The expression of antioxidant enzymes was also up-regulated when Arabidopsis was treated with OTA [37]. Superoxide dismutase converts ROS to H_2_O_2_, thereby limiting the potential toxicity of ROS, and is an important antioxidant metalloenzyme in organisms [40,41]. The present work shows that the expression of the SOD gene secreted by the ML17 strain is markedly up-regulated after OTA treatment, which contributes to the scavenging of ROS and protects cells from oxidative stress damage. Genes related to antioxidant activity, such as peroxisomes, SOD, and GSH3, were significantly upregulated in *B. naejangsanensis* ML17 after exposure to OTA, suggesting that ML17 strain can resist the toxic effects of OTA through regulation of its metabolism, and enzyme genes with OTA-degrading activity (*gene0095*) were found to be upregulated. To summarize, we suggest that after OTA treatment, *B. naejangsanensis* ML17 induces systemic regulation of the redox network by upregulating the expression of specific antioxidant enzymes, which reduces cellular damage caused by OTA.

**Figure 6:**
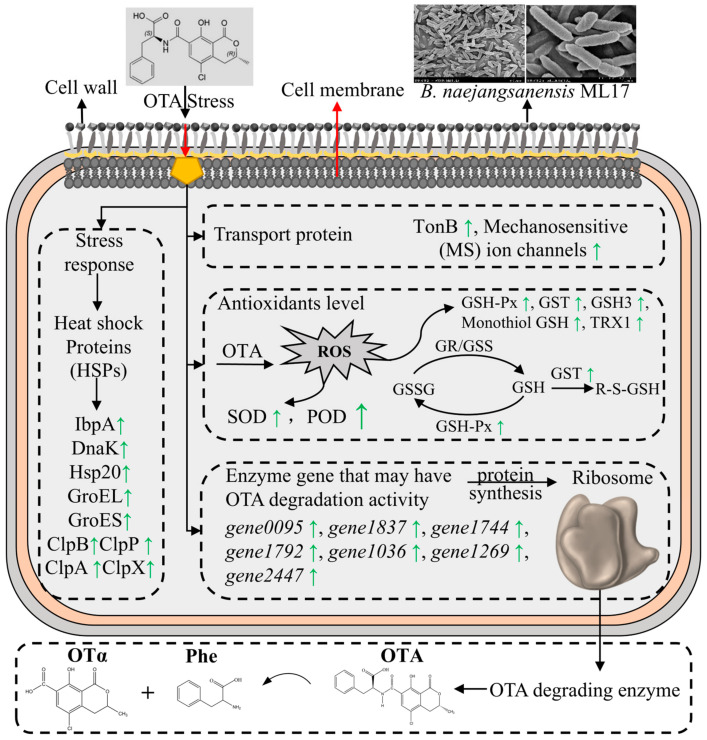
Schematic diagram of the OTA degradation mechanism in *B. naejangsanensis* ML17. Green arrows represent significant increases in gene expression.

Combining the results of previous studies, the transcriptomic analysis of *B. naejangsanensis* ML17 under OTA stress, and the OTA degradation effect of its stress enzymes, we speculate that the degradation of OTA by *B. naejangsanensis* ML17 is the result of the joint action of multiple factors (Figure 6). In the presence of OTA, *B. naejangsanensis* ML17 rapidly upregulated oxidative stress-related pathway genes to resist the toxic effects of OTA. This made the effect on the growth of *B. naejangsanensis* ML17 insignificant in the presence of lower concentrations of OTA. However, when the toxin concentration was too high, the toxic effect of OTA on the strain was enhanced, resulting in the slow growth of *B. naejangsanensis* ML17 and increased morphological abnormalities in the organism. At the same time, *B. naejangsanensis* ML17 upregulated the expression of an OTA-degrading enzyme to degrade OTA into non-toxic OTα, thereby reducing the toxic effect of OTA on itself.

## 5. Conclusions

*B. naejangsanensis* ML17 showed a very high tolerance to OTA. The growth of ML17 was not affected by the exposure to 6 μg/mL of OTA. Transcriptomics analysis showed that genes related to antioxidant activity, such as peroxidase, SOD, and GSH3, were significantly upregulated. Meanwhile, the *gene0095*-encoding enzyme showed OTA-degrading activity; *gene1826*, *gene2253*, *gene0484*, *gene1837*, *gene1744*, *gene1792* and *gene1036* gene-encoding enzymes may also have OTA-degrading activity which need further research. This suggests that ML17 can degrade OTA by regulating its metabolism, upregulating its antioxidant system, and upregulating the expression of enzyme genes with OTA-degrading activity.

## Figures and Tables

**Table 1:** Expression of heat-shock proteins in *B. naejangsanensis* under OTA stress.

Gene ID	log_2_(Test/Control)	−log_10_(*p* Adjust)	Gene Name (EC Number)	COG/GO Description
*gene0023*	2.916	1.941	Heat-shock protein/molecular chaperone IbpA, Hsp20	Response to heat
*gene0103*	3.243	2.395	Heat-shock protein/molecular chaperone DnaK, Hsp20	Heat shock protein
*gene0607*	2.519	2.082	Hsp20/alpha crystallin family	Response to heat
*gene0291*	2.901	2.288	Chaperonin GroEL	Prevents misfolding under stress conditions
*gene0292*	3.928	2.303	Chaperonin GroES	Binds to cpn60 in the presence of Mg-ATP
*gene2710*	2.130	1.919	ATP-dependent chaperone ClpB	ATP-dependent CLP protease ATP-binding subunit
*gene1265*	1.900	1.394	ATP-dependent ClpP protease, Protease subunit [EC:3.4.21.92]	Cleaves peptides in various proteins in a process that requires ATP hydrolysis.
*gene1268*	2.392	1.578	ATP-binding subunit ClpX	ATP-dependent specificity component of the Clp protease.
*gene1075*	1.992	1.775	ATP-binding subunit ClpA	ATP-dependent CLP protease ATP-binding subunit
*gene0971*	3.008	1.791	Stress-induced protein	Stress-induced protein, KGG, repeat protein
*gene2217*	2.483	2.221	Universal stress protein	Universal stress protein
*gene0017*	3.770	2.695	GlsB/YeaQ/YmgE family stress response membrane protein	Integral component of membrane

Note: log_2_(test/control) > 1, −log_10_(*p*-adjusted) > 1.301 indicates a significant difference.

**Table 2:** Expression of transport proteins in *B. naejangsanensis* under OTA stress.

Gene ID	log_2_(Test/Control)	−log_10_(*p* Adjust)	Gene Name (EC Number)	COG/GO Description
*gene2071*	3.788	2.951	Biopolymer transport protein ExbD	Biopolymer transport protein exbD tolR
*gene2072*	2.428	1.674	Biopolymer transport protein ExbB	MotA TolQ exbB proton channel
*gene2070*	2.243	1.885	periplasmic protein TonB	TonB family
*gene2485*	2.877	2.403	TonB-dependent receptor	tonB-dependent Receptor
*gene0900*	2.125	1.530	TonB-dependent receptor	tonB-dependent Receptor
*gene1458*	1.503	1.593	vitamin B_12_ transporter	tonB-dependent Receptor
*gene0063*	1.844	1.572	Iron complex outermembrane recepter protein	Receptor
*gene1933*	1.484	1.606	Periplasmic copper chaperone A	Secreted protein
*gene0998*	2.838	1.562	porin family protein	Outer membrane protein beta-barrel domain
*gene1393*	1.434	1.437	Outer membrane protein insertion porin family	Gram-negative-bacterium-type cell outer membrane assembly
*gene0860*	1.350	1.462	Sodium–solute symporter family	Symporter
*gene0013*	1.570	1.305	Putative sulfate exporter family transporter	Membrane
*gene1088*	1.428	1.488	Mechanosensitive ion channel	Mechanosensitive ion channel

Note: log_2_(test/control) > 1, −log_10_(*p*-adjusted) > 1.301 indicates a significant difference.

**Table 3:** Expression of enzymatic antioxidants in *B. naejangsanensis* under OTA stress.

Gene ID	log_2_(Test/Control)	−log_10_(*p* Adjust)	Gene Name (EC Number)	COG/GO Description
*gene1531*	2.122	2.343	Peroxiredoxin Q/BCP [EC:1.11.1.15]	Alkyl hydroperoxide reductase Thiol specific antioxidant Mal allergen
*gene0243*	2.445	1.807	Peroxiredoxin (alkyl hydroperoxide reductase subunit C) [EC:1.11.1.15]	Alkyl hydroperoxide reductase
*gene0016*	2.103	2.085	Superoxide dismutase	Superoxide dismutase
*gene2362*	2.773	2.320	Superoxide dismutase, Fe-Mn family [EC:1.15.1.1]	Destroys radicals which are normally produced within the cells and which are toxic to biological systems
*gene1643*	1.574	1.431	Glutathione peroxidase [EC:1.11.1.9]	Glutathione peroxidase
*gene1765*	1.428	1.383	Glutathione S-transferase [EC:2.5.1.18]	Glutathione S-transferase
*gene1263*	1.102	1.575	Hydroxyacylglutathione hydrolase [EC:3.1.2.6]	Beta-lactamase domain protein
*gene0415*	1.944	1.575	Glutaredoxin 3	Glutaredoxin
*gene2353*	1.972	1.431	Monothiol glutaredoxin	Glutaredoxin
*gene1226*	4.108	1.714	tRNA-Glu	tRNA-Glu
*gene1227*	3.694	2.255	tRNA-Glu	tRNA-Glu
*gene2054*	3.179	1.475	tRNA-Gly	tRNA-Gly
*gene2055*	3.209	2.395	tRNA-Tyr	tRNA-Tyr
*gene1697*	1.274	1.576	tRNA-Arg	tRNA-Arg

Note: log_2_(test/control) > 1, −log_10_(*p*-adjusted) > 1.301 indicates a significant difference.

**Table 4:** Differential expression of active enzymes in *B. naejangsanensis* ML17 under OTA stress.

Gene ID	log_2_(Test/Control)	−log_10_(*p* Adjust)	Gene Name (EC Number)	COG/GO Description
*gene0095*	1.404	1.530	Peptidyl-dipeptidase Dcp [EC:3.4.15.5]	Metal ion binding, metalloendopeptidase activity
*gene1826*	0.060	0.072	Serine carboxypeptidase	Serine-type carboxypeptidase activity
*gene2253*	−0.071	0.117	M14 family metallopeptidase	Zinc ion binding, metallocarboxypeptidase activity
*gene0484*	−0.222	0.286	Amidohydrolase family protein	Hydrolase activity, acting on carbon-nitrogen (but not peptide) bonds
*gene1837*	1.706	1.475	Leucyl aminopeptidase [EC:3.4.11.1]	Cytoplasm, manganese ion binding, aminopeptidase activity, metalloexopeptidase activity
*gene1744*	1.587	1.357	Peptidase	Bacterial pre-peptidase C-terminal domain
*gene1792*	1.521	1.540	Serine hydrolase/*β*-lactamase	Integral component of membrane, hydrolase activity
*gene1036*	1.542	1.395	Do family serine Endopeptidase/trypsin-like peptidase	Periplasmic space; serine-type endopeptidase activity

Note: log_2_(test/control) > 1, −log_10_(*p*-adjusted) > 1.301 indicates a significant difference.

## Data Availability

The original contributions presented in the study are included in the article/Supplementary Materials, further inquiries can be directed to the corresponding author.

## Supplementary Materials

The following supporting information can be downloaded at: https://www.mdpi.com/article/10.3390/foods13233732/s1, Table S1: All co-expressed genes in the control and experimental groups; Table S2: Genes with significant differences in expression in the experimental group; Table S3: Functional enrichment analysis of differentially expressed genes.
